# Effectiveness of a Mobile Health Application for Educating Outpatients about Bowel Preparation

**DOI:** 10.3390/healthcare12141374

**Published:** 2024-07-10

**Authors:** Hui-Yu Chen, Ming-Hsiang Tu, Miao-Yen Chen

**Affiliations:** 1 School of Nursing, National Taipei University of Nursing and Health Sciences, Taipei 112303, Taiwan; hychen30@vghtpe.gov.tw (H.-Y.C.); miaoyen@ntunhs.edu.tw (M.-Y.C.); 2 Endoscopy Center for Diagnosis and Treatment, Taipei Veterans General Hospital, Taipei 112201, Taiwan

**Keywords:** colonoscopy, bowel preparation, outpatient, nursing

## Abstract

Colonoscopy is an essential method for diagnosing and treating colorectal cancer, relying on effective bowel preparation to thoroughly examine the large intestinal mucosa. Traditional education involves printed instructions and verbal explanations but does not guarantee clear patient understanding. Poor bowel preparation can obscure mucosal visibility, delaying cancer diagnosis and treatment. A mobile medical model using Android devices for bowel preparation education was tested in a single-blind, randomized trial. This trial enrolled outpatients undergoing colonoscopy at the Endoscopy Center for Diagnostic and Treatment between 27 October 2021 and 31 December 2022. This study introduced the ColonClean app alongside traditional methods. After examination, endoscopists rated the preparation quality using the Aronchick scale. A data analysis was conducted using SPSS 25.0 to determine if there was a significant improvement in bowel preparation quality between the control group (traditional method) and the experimental group (traditional method plus the ColonClean app). Forty patients were recruited in each group. In the experimental group, all ratings were “fair”, with 75% receiving an “excellent” or “good” rating, showing statistical significance (*p* = 0.016). The ColonClean app improves bowel preparation quality more effectively than traditional care instructions.

## 1. Introduction

Colonoscopy has advantages in diagnosing, screening, and treating colorectal lesions, as it allows for direct sampling and resection of lesions, thereby preventing adenomatous polyps from developing into cancer. It is an accurate and well-tolerated examination method [1,2,3]. Screening through colonoscopy can prevent colorectal cancer, which ranks third in global incidence and second in mortality rates [4]. Early-stage colorectal cancer often presents no symptoms, leading to overlooked warning signs [5,6]. The World Health Organization has stated that 30%–50% of cancer cases are preventable, highlighting the importance of preventive measures [7,8].

Bowel preparation before colonoscopy involves dietary restrictions and using bowel-cleansing agents to remove fecal matter from the colon, providing optimal conditions for mucosal visualization during the procedure [9,10]. This step is crucial for the overall integrity of colonoscopy [7,11]. An incomplete colonoscopy prevents adequate visualization of the colonic mucosa, hampers the detection of lesions, and delays cancer diagnosis and treatment [12].

Bowel preparation quality is assessed during colonoscopy using the Aronchick Bowel Preparation Scale, which evaluates the percentage of colonic mucosa covered by feces [13]. A thorough bowel preparation provides the physician with a clear view of the entire colonic mucosa during the examination, enabling the identification of polyps or other abnormalities. This enhanced visibility contributed to a 5% increase in adenoma detection rates [14]. Conversely, inadequate bowel preparation may lead to missed pathological findings, prolonged procedure times, failed detection of cancerous lesions, and increased risk of adverse events during the examination [15,16,17].

Traditional methods of bowel preparation education before colonoscopy involve providing printed care instruction sheets accompanied by verbal explanations. However, conveying bowel preparation instructions solely through text and oral communication may not ensure patients’ clear understanding. Complex and lengthy instructions can lead to poor adherence, with up to 25%–30% of patients achieving inadequate bowel preparation [14]. Poor bowel preparation is associated with a high rate of missed polyps, particularly sessile polyps on the colonic mucosa. Therefore, improving bowel preparation quality is crucial [7,11,18].

The World Health Organization defines mobile health as using wireless communication devices to support public health and clinical practice, recognizing it as a safe and cost-effective care method [19,20]. Smartphone apps have become increasingly popular in healthcare, offering solutions to many traditional healthcare challenges. With the advent of the digital and mobile era, mobile health apps have emerged as an important trend in the healthcare industry, offering various lifestyle and health management tools such as smoking cessation support and exercise tracking.

Effective nursing guidance can provide planned nursing behaviors, aiding patients in understanding and addressing health issues. Besides conventional methods using printed care instruction sheets, various approaches such as educational booklets, videos, images, telephone calls, and text message reminders have been used to improve bowel preparation efficacy. However, integrating smartphone apps with verbal and printed nursing guidance education can further enhance bowel preparation efficacy by 10% [21,22]. Regardless of the method used, the goal is to improve the quality of bowel preparation.

Following the COVID-19 pandemic and the need to reduce virus transmission by maintaining physical distance, mobile health apps have become even more critical [23,24]. During current public health emergencies, using personal communication tools to provide remote medical services has become essential [25,26]. Using mobile devices in conjunction with mobile apps to assist traditional printed nursing guidance, supplemented with verbal instructions for bowel preparation, can enhance the level of bowel preparation. Using mobile apps to reinforce patient education on bowel preparation can improve the overall quality of bowel preparation [17,27].

In this study, a mobile health app called ColonClean was designed using mobile devices and information and communication technology. It was developed on the Android operating system as a nursing guidance tool for bowel preparation. This approach provides a safe and cost-effective method of guidance. Bowel preparation requirements are complex and challenging to understand and remember. Effective use of reminders, food diagrams, step-by-step illustrations of bowel-cleansing medication, and audiovisual aids can enhance patient understanding of bowel preparation. Reference images of fecal characteristics also help patients to understand whether their bowel movements meet the requirements for colonoscopy, increasing patient compliance.

This study aims to evaluate the effectiveness of a mobile healthcare model using a mobile application for bowel preparation education in colonoscopy. Endoscopists require a clean and intact mucosa for optimal visualization during the examination or treatment of lesions within the intestine. To achieve this, patients are provided with instructions, including dietary restrictions and the use of bowel cleansers, to clear the colon of fecal matter, ensuring the best conditions for mucosal visualization during the procedure. The completeness of colonoscopy is crucial for adequately visualizing the colonic mucosa, detecting lesions, and timely diagnosis and treatment of cancer.

This study combines the design of a mobile application with traditional oral and printed care instructions to enhance patients’ understanding and adherence to bowel preparation. We will investigate whether the mobile application can effectively improve patients’ compliance with bowel preparation and compare its efficacy with traditional instruction methods in improving the quality of bowel preparation. We hope this study will demonstrate the potential of mobile applications in enhancing bowel preparation education and facilitating comprehensive colonoscopies, ultimately contributing to reduced healthcare costs.

## 2. Materials and Methods

### 2.1. Study Procedures

This study is an interventional investigation, and according to research ethics regulations, it is essential to understand and comply with these norms when conducting research. An approval from the Institutional Review Board of the Taipei Veterans General Hospital has been obtained prior to commencement of this study (approval number: 2021-10-007AC). It was conducted from 27 October 2021 to 31 December 2022. Individuals were enrolled in this study after its purpose and content had been explained to them, and they had provided consent by signing an informed consent form.

### 2.2. Study Design

This study was conducted in the outpatient clinics of the Endoscopy Center for Diagnostic and Treatment in Northern Taiwan. It adopted a quantitative research approach, using a single-blind, randomized, quasi-experimental design with a post-test-only, equivalent-group design. In the single-blind study design, the endoscopists performing the colonoscopy do not know whether the study subjects are from the experimental group or the control group.

Based on the reference research design [28], a sample size of 64 individuals was estimated for this study using the G*Power for Windows software (version 3.1.9.7), considering a statistical power (1 − β) of 0.8 and an α value of 0.05, with a moderate effect size of 0.3 due to the importance of bowel preparation. Accounting for a dropout rate of 20% to compensate for potential sample loss and invalid data, it was decided that this study should include 80 participants, with 40 allocated to each of the experimental and control groups.

**Figure 1:**
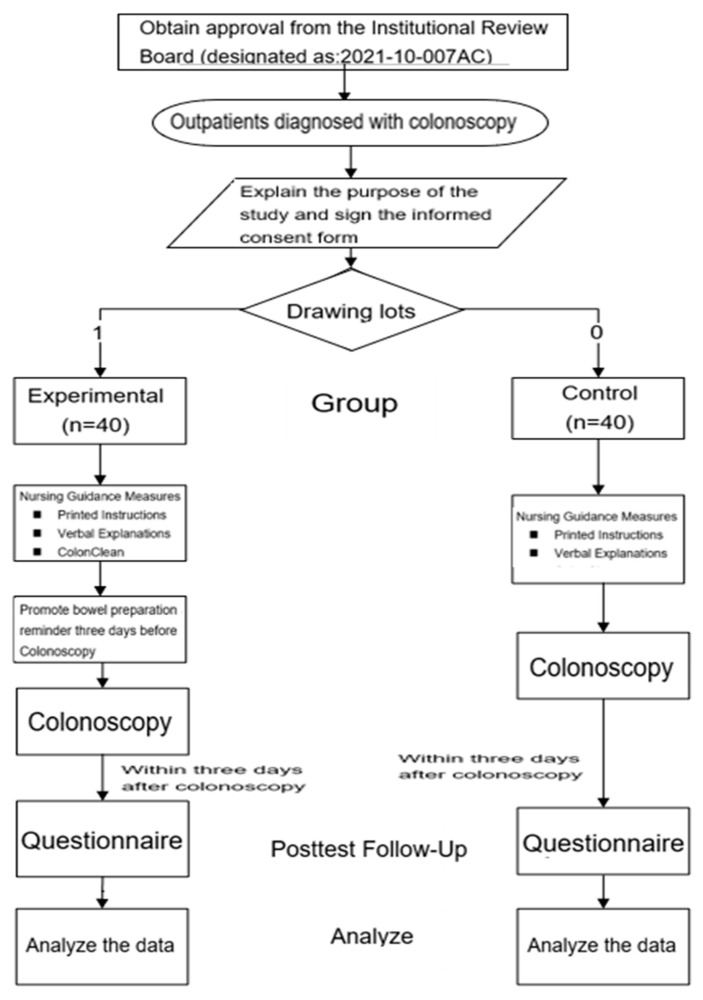
Research design flowchart.

Two sheets of paper with 0 and 1 written on them were prepared to allocate participants to the respective groups. Participants drew one of these sheets to determine their group assignment. Those who drew the sheet with the number 0 were assigned to the control group, while those who drew the sheet with the number 1 were assigned to the experimental group. After the participants were included in this study, both groups completed the basic data questionnaire within 3 days after the colonoscopy (Figure 1).

### 2.3. Population

Individuals recommended by their physicians to undergo colonoscopy had to meet the following criteria:

1. Aged ≥ 20 years.
2. Able to communicate in Taiwanese or Mandarin.
3. No visual or hearing impairments.
4. Own an Android-based mobile device.
5. No severe mental or cognitive impairments and able to comply with the study requirements.

### 2.4. Research Tool (Mobile Health App)

Regarding the precautions for bowel preparation before colonoscopy, the paper-based nursing guidance leaflet includes the purpose of colonoscopy, precautions for using bowel preparation drugs, dietary preparation, and precautions after colonoscopy. The control group used conventional paper-based nursing instruction leaflets combined with oral instructions. In addition to the printed care instructions and verbal instructions, the experimental group also used the ColonClean app as an intervention. The ColonClean app was developed using the Android 11 operating system. The user interface and content were designed using the Android application package, Java programming language, and a built-in database. A team consisting of a clinical nurse, a user interface designer, and an information engineer developed the app. The design was based on an assessment of current needs and supported by a literature review.

Participants in the experimental group downloaded the ColonClean app to their smartphones or mobile devices through the provided QR code. The app has a dedicated icon on its home screen for easy access. After receiving instructions from the researchers on how to use the app, the participants in the experimental group entered their personal information, their exam date, and the details of the bowel preparation medication they would use.

Patients in both groups received one-on-one instruction during the nursing guidance process. These instructions were provided in a dedicated waiting area at the outpatient clinic before their appointment and continued until the participant fully understood the information provided. The ColonClean app was designed for the Android operating system. Its content was identical to the printed nursing instructions [29], but it enhanced users’ understanding by replacing text descriptions with cartoon images and step-by-step visuals. For example, dietary choices, instructions for bowel preparation medication use, and other relevant information are presented using cartoon images and visual flowcharts to facilitate better understanding. The app includes a feature for recording water intake after taking bowel preparation medication to promote the effectiveness of bowel preparation by helping users ensure they consume sufficient water.

**Figure 2:**
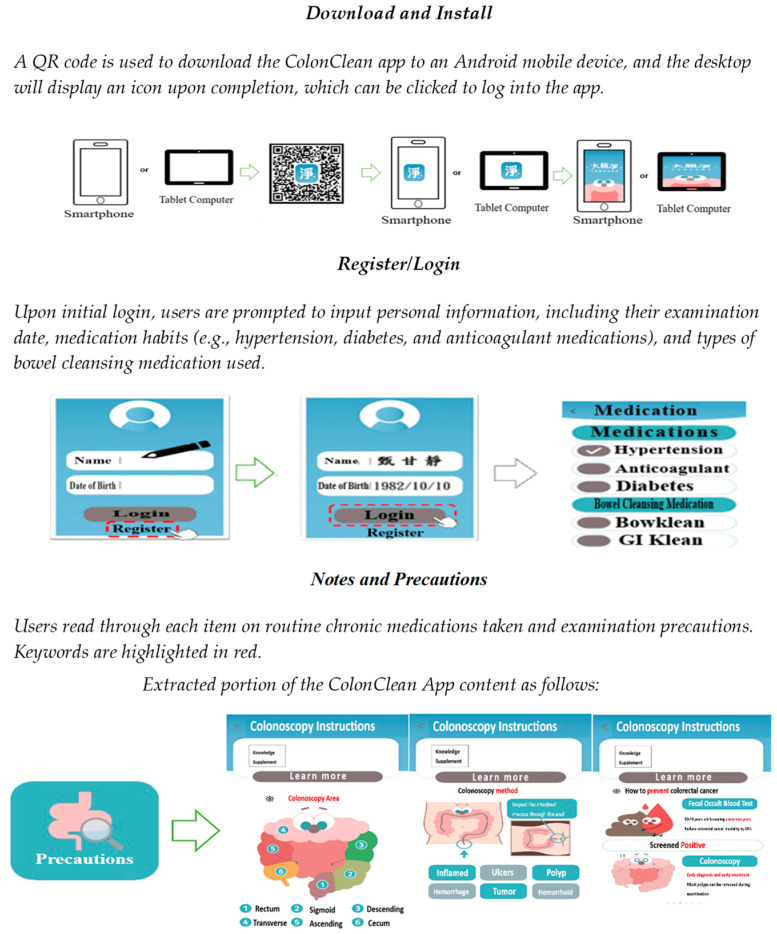
The content of the ColonClean app.

The app also guides interpreting the last bowel movement, including suggestions based on images of different types of bowel movements and a visualization of the level of bowel preparation for colonoscopy. All aspects of bowel preparation are also presented in a dedicated video section, allowing users to understand the process through audiovisual means (Figure 2).

Participants in the control group had to be mindful of the starting time for bowel preparation. In contrast, the ColonClean app provided reminders and notifications to participants in the experimental group starting three days before their examination. These reminders suggest initiating dietary restrictions, such as avoiding high-fiber fruits and vegetables. Two days before the examination, the app reminds users to switch to a low-residue diet (e.g., congee and white noodles). On the day before the examination, the app notifies users to follow a clear liquid diet (e.g., oil-free broth and sports drinks) and provides instructions on how to use bowel preparation medications.

During the bowel preparation period, users can use the app to access information on dietary choices, recommendations for water intake, the importance of bowel preparation, and reference images for interpreting the last bowel movement. The app also provides audiovisual content to help users understand how to use bowel preparation medications. On the day of the examination, participants in the experimental group could take a photo of their last bowel movement and compare it with the built-in images in the ColonClean app to assess whether their bowel preparation quality met the requirements of excellent, good, fair, or poor. If participants encounter any issues during the bowel preparation process, they can refer to the app’s built-in “Notes and Precautions” section for assistance.

### 2.5. Endoscopists’ Qualifications

To ensure consistency in assessing the bowel preparation quality in this study, both endoscopists performing colonoscopies possessed qualifications as specialist physicians in gastrointestinal endoscopy and were attending physicians. They had at least one year of service at the gastroenterology and endoscopy center and performed over 500 colonoscopies yearly. In the single-blind study design, the endoscopists were unaware of the group assignments of the study participants. The endoscopists evaluated the effectiveness of bowel preparation in the study subjects using the Aronchick Bowel Preparation Scale [18].

### 2.6. Statistical Analysis

The data were analyzed using SPSS software (version 25.0). The number and percentages of participants in each category of the Aronchick Bowel Preparation Scale were compared between the experimental and control groups using a chi-square test to determine whether bowel preparation quality, as judged by endoscopists, differed significantly between them.

## 3. Results

### 3.1. Participants’ Characteristics

This study uses the mean, standard deviation, number of people, and percentage distribution to analyze the differences in the basic demographic information of the two groups of research subjects. This study included 80 participants who were randomly assigned to either the experimental (*n* = 40) or control (*n* = 40) group. All 80 participants completed colonoscopy without any mid-process cancellations or exclusions due to missing data. The two groups differed significantly only in body mass index (BMI; *p* = 0.020). The difference in BMI between the two groups was approximately 2.5. The participants’ demographic characteristics were as follows: 51.2% were male, 30% were aged 51–60 years, their mean BMI was 23.79 ± 3.97, their highest educational attainment was a college/university degree (45%), most were married (76.25%), 21.25% reported constipation, 35% had a history of abdominal surgery, 15% had a history of diabetes, 17.5% had a family history of colorectal cancer, and 60% had previous experience with colonoscopy (Table 1).

### 3.2. Bowel Preparation Quality

This study compared the frequency and percentage of bowel preparation quality determined by endoscopists using the Aronchick Bowel Preparation Scale between groups using a chi-square test (Table 2). The endoscopists assessed the bowel preparation quality according to the Aronchick Bowel Preparation Scale during the colonoscopy procedure, classifying it as “excellent”, “good”, “fair”, or “poor”, and documenting it in the colonoscopy report after the examination. In the experimental group, 10 participants (25%) were rated as fair, 26 (65%) as good, and 4 (10%) as excellent. In the control group, 7 participants (17.5%) were rated as poor, 13 (32.5%) as fair, 19 (47.5%) as good, and 1 (2.5%) as excellent. The chi-square test indicated a significant difference between groups (*p* = 0.016). Based on the chi-square test, there was a significant association between the bowel preparation quality assessments in the experimental and control groups and the judgments made by endoscopists (χ^2^ = 16.945, df = 3, *p* = 0.016 *), with a moderate effect size observed (Cramer’s V = 0.46). This suggests that differences in bowel preparation quality between the experimental and control groups may significantly influence endoscopists’ judgments. The experimental group utilized both paper and verbal nursing measures along with the ColonClean app, whereas the control group used only paper and verbal nursing instructions. The quality of bowel preparation was assessed by endoscopists through colonoscopy. In the experimental group, no subjects were rated as having poor bowel preparation, with 30 subjects (75%) rated as good or excellent, compared to 20 subjects (50%) in the control group. This indicates that the use of the ColonClean app resulted in better bowel preparation as judged by endoscopists compared to using only paper and verbal nursing measures.

**Figure 3:**
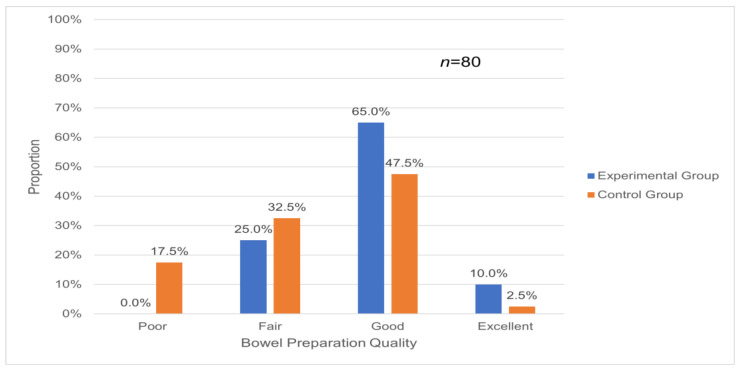
Endoscopists’ assessment of bowel preparation quality.

It investigated whether bowel preparation quality, as judged by endoscopists, differed significantly between the experimental and control groups (Figure 3).

The endoscopists’ assessments of bowel preparation quality showed the following distribution:

- “Excellent” preparation: higher in the experimental group (10%) than in the control group (2.5%).
- “Good” preparation: higher in the experimental group (65%) than in the control group (47.5%).
- “Fair” preparation: lower in the experimental group (25%) than in the control group (32.5%).
- “Poor” preparation: absent in the experimental group (0%) and present in the control group (17.5%).

Analysis of the two groups revealed a predominance of yellow watery stool characteristics reported during the last bowel movement, consistent with Shin et al. (2019) [30], which suggests suitability for colonoscopy examination based on stool attributes.

This study’s results demonstrate that bowel preparation quality was significantly superior in the experimental group compared to the control group. This finding has significant implications for clinical nursing guidance and practice, suggesting that a mobile healthcare model can effectively assist individuals in achieving better bowel preparation before endoscopic examinations. This improvement can potentially enhance the efficiency and accuracy of examinations, reduce procedural complexity, increase patient satisfaction, and possibly lower the risk of complications and adverse events associated with the procedure.

This research used the ColonClean app to educate users on bowel preparation effectiveness, as assessed by endoscopists using the Aronchick Bowel Preparation Scale. While the “poor” category was not observed, the proportions of “excellent” and “good” ratings did not meet the domestic colonoscopy quality target of over 90% [31].

The quality of bowel preparation and attainment of complete cecal intubation are indispensable components of a comprehensive and successful colonoscopy procedure. Complete cecal intubation is a vital metric in evaluating colonoscopy screening quality, involving the insertion of the colonoscope near the ileocecal valve to thoroughly examine the cecum, including the intermediate area between the appendiceal orifice and the ileocecal valve. As approximately 15% of all polyps and cancerous tumors are located in the cecum, a cecal intubation rate of ≥90% is recommended to facilitate the detection of hidden lesions, enabling effective further diagnosis and treatment [32,33,34].

The cecal intubation rate was 100% among participants in both the experimental and control groups, with no interruptions or cancellations of colonoscopy procedures. The participants found the bowel preparation process to be strenuous. To facilitate the successful completion of colonoscopy examinations, the study hospitals used a Flushing Pump OFP-2 during the procedures. Physicians often use this equipment to cleanse the colon when the quality of bowel preparation allows for the flushing and suctioning of fecal matter and debris. This approach yielded outcomes consistent with scholarly findings, demonstrating complete cecal intubation rates. Participants undergoing colonoscopy examinations endured the challenges associated with bowel preparation. The study hospitals aimed to mitigate the rate of incomplete colonoscopy examinations and reduce the likelihood of repeat procedures by leveraging professional-grade equipment [35].

## 4. Discussion

The effectiveness of different nursing instruction methods on bowel preparation is crucial. For outpatient cases, understanding the importance of bowel preparation before colonoscopy and the procedure itself is pivotal for a successful examination [36]. Difficulty understanding the requirements for bowel preparation or misconceptions about its significance during the first colonoscopy can lead to inadequate preparation, affecting the effectiveness of the examination and potentially masking underlying conditions [37,38]. When the proportion of inadequate bowel preparation exceeds 10%–15%, it is necessary to reassess the preparation plan and implement improvement measures. Updated methods of bowel preparation guidance can further educate patients on adhering to pre-preparation instructions, enhancing the likelihood of achieving optimal intestinal cleansing [39,40,41]. However, relying solely on paper-based nursing instruction sheets and verbal communication is insufficient. In addition to conventional methods, it is recommended that healthcare professionals use various educational strategies to achieve a more comprehensive level of bowel preparation [41].

Enhanced education by healthcare professionals significantly improves compliance with bowel preparation. For colonoscopy, educational pamphlets may serve as relevant, cost-effective, and valuable materials. Healthcare professionals explain the bowel preparation process clearly to patients during outpatient visits and provide pamphlets with clear, color-coded illustrations as reinforcement tools for verbal communication [42]. The pamphlets emphasize dietary restrictions, the importance of adequate hydration, and the potential side effects of bowel preparation medications. A previous study found that an enhanced patient education program significantly increased the level of bowel preparation for colonoscopy, with a significantly higher proportion of excellent preparation in the intervention group than in the control group (98.7% vs. 52.3%, *p* < 0.001) [42].

Another study [43] recruited 657 research participants aged 18–85 years with a history of failed bowel preparation from 11 hospitals in Spain who were randomly assigned to either the control group, which received paper-based nursing instruction sheets and verbal explanations, or the intervention group, which received additional reinforcement through telephone education by nurses within 48 h before their colonoscopy. The primary goals of the enhanced nursing instruction were to ensure compliance with dietary restrictions and intake of bowel preparation medications, emphasize the importance of proper bowel preparation, and address any concerns or doubts during the preparation process. Its results showed no significant difference in the level of bowel preparation between the two groups (77.3% vs. 72%, *p* = 0.12). However, there was a trend towards more adequate bowel preparation, with an improvement of approximately 11.5%. There was also a trend towards improved colonoscopy completion rates among participants who received nurse-led telephone education. These findings suggest that telephone reinforcement education by nurses may be an effective tool for improving bowel preparation.

In another study, nurses provided patients with a colonoscopy information booklet for bowel preparation education, covering dietary arrangements, methods of using bowel preparation medications, and the colonoscopy procedure [44]. In addition to verbal instructions, patients received two video SMS messages on their smartphones. Its results indicated that educational video SMS messages delivered via smartphones led to better bowel preparation, serving as a tool to enhance the safety and effectiveness of colonoscopy. However, this educational method may be more beneficial for individuals aged under 40 years who are likely more frequent and proficient smartphone users than older adults.

Another approach involves using paper-based nursing instruction sheets with vivid illustrations and simple, understandable text supplemented by verbal explanations. Information related to the paper-based content was also sent through the WeChat mobile social media application, providing online real-time assistance for bowel preparation guidance. The results showed a 90.1% completion rate of bowel preparation. When comparing the reinforcement measures provided through WeChat and SMS, WeChat was found to be superior to SMS in achieving optimal levels of bowel preparation [45,46].

Mobile health apps represent a novel educational tool that can facilitate better and more comprehensive bowel preparation before colonoscopy. In outpatient settings, patients receive push notifications starting four days before the colonoscopy, providing visual aids of low-residue and clear liquid diets, reminders on when to start consuming appropriate diet types, and notifications regarding the use of bowel preparation medications. These apps feature visual aids and reminders, resulting in 92.3% of users achieving optimal levels of bowel preparation, with 60.6% reaching excellent levels [17,47].

In this study, the ColonClean app began sending reminders to start bowel preparation three days before the colonoscopy, along with recommendations on dietary restrictions. During the preparation period, users can access text and image-based guidance on dietary choices, recommended fluid intake, the importance of intestinal cleansing, and reference materials on the characteristics of the last bowel movement. Video references are also available to help understand how to use bowel-cleansing medications. On the day of the examination, users take a photo of their last bowel movement and compare it with the images built into the ColonClean app to assess whether the quality of their bowel preparation meets the criteria of excellent, good, fair, or poor. As a supportive tool for bowel preparation guidance, the ColonClean app effectively increased the proportion of excellent and good preparation levels to 75%. The quality of bowel preparation and the achievement of complete cecal intubation rates are indispensable parts of comprehensive and successful colonoscopy. Although the proportion of bowel preparation rated as “excellent” and “good” did not meet the domestic quality target for colonoscopy of over 90% [31], the experimental group did not observe any “poor” ratings. This indicates that adopting the mobile health care model in this study can effectively help individuals achieve better bowel preparation before colonoscopy. The cecal intubation rate is a crucial indicator for assessing colonoscopy quality, with a recommended rate of ≥90% to facilitate the detection of hidden lesions, enabling effective diagnosis and treatment [32,33,34]. In this study, the cecal intubation rate was 100% in both groups, with no interruptions or cancellations of the colonoscopy. Although it is unclear whether the differences between the two groups are related to the measures adopted, it emphasizes the necessity of enhanced education. When the quality of bowel preparation allows for the flushing and suction of feces and debris, physicians will use the Flushing Pump OFP-2 to clean the colon, reducing the incidence of incomplete colonoscopy and the likelihood of repeat procedures [35]. This improves the efficiency and accuracy of colonoscopy, reduces procedural complexity, enhances patient satisfaction, and potentially lowers the risk of procedure-related complications and adverse events.

It is believed that compared to methods such as phone calls or SMS, the ColonClean app can also reduce the workload of healthcare personnel, making it more suitable for the current COVID-19 pandemic situation in our country. Regardless of the method used, it should align with the culture and requirements at the time, aiming to achieve optimal benefits at a minimal cost [48].

This study has several study limitations. First, this study was only conducted at one medical center in Northern Taiwan. While the ColonClean app was used as a tool for bowel preparation nursing guidance, variations in the choice and use of bowel-cleansing medications among different medical institutions and differences in physicians’ medication preferences may exist. It is recommended that future studies be conducted at various medical institutions to confirm whether nursing guidance programs for bowel preparation before colonoscopy, assisted by mobile healthcare models, yield superior effectiveness. Second, the ColonClean app developed in this study was designed explicitly for the Android operating system on mobile devices. It does not support other operating systems. It is suggested that future research develop mobile healthcare apps compatible with all major operating systems to serve as comprehensive nursing guidance tools for bowel preparation. Third, the proportion of participants with a college degree or higher was 72.5% in the experimental group, compared to only 50% in the control group. This educational disparity between groups should be acknowledged as a potential limitation of this study.

This study has several suggestions. First, while the ColonClean app provides users with cartoon images, videos, and other means to understand bowel preparation, it is suggested that real-time interactive features be added to address any issues encountered during the preparation process. Their inclusion would effectively stabilize users’ emotions during preparation and reduce concerns about incomplete bowel preparation. Second, in response to the advent of artificial intelligence (AI), it is recommended that AI recognition functionality be integrated into the ColonClean app to understand the nature of the last bowel movement [49]. Using AI, the app could automatically identify and determine the attributes of the last bowel movement, which would provide analysis and effective recommendations for individuals with poor bowel preparation quality, thereby enhancing the overall quality of bowel preparation [50,51]. Third, for future optimization of the ColonClean app, it is suggested that it be promoted or listed on various app store platforms to make it accessible to a wider audience. By offering compatibility with different operating systems and devices, the app could be made available to individuals needing bowel preparation, thereby benefiting a larger population.

## 5. Conclusions

Early-stage colorectal cancer is often asymptomatic and can easily go unnoticed. Colonoscopy can identify colorectal diseases, precancerous lesions, or malignant tumors to allow for further diagnosis and treatment, thereby enabling early detection and intervention to reduce the incidence and mortality of colorectal cancer. During the colonoscopy, endoscopists can directly sample lesions and remove adenomatous polyps, preventing their progression to colorectal cancer. Adequate bowel preparation before the colonoscopy is crucial for the integrity of the examination. Fecal matter is removed from the colon through a dual strategy of dietary restrictions and bowel-cleansing medications, providing a clear view of the colonic mucosa for the endoscopists’ assessment.

Traditionally, bowel preparation guidance has relied on paper-based instructions supplemented by verbal explanations. However, using the ColonClean app for pre-colonoscopy bowel preparation guidance has enhanced users’ understanding of dietary concepts, such as clear liquid, low-residue, and high-fiber diets, through visual aids. Push notifications remind users of dietary, medication, and appointment timing, enhancing the accuracy and compliance of bowel preparation. The app effectively assists individuals in completing bowel preparation tasks by adopting a user-centered approach.

This study provides a mobile health care model to assist with bowel preparation guidance before outpatient colonoscopy. The results show that the ColonClean app enhances the effectiveness of bowel preparation compared to traditional guidance methods. This implies that the mobile health care model is an effective approach for achieving high-quality bowel preparation and improving the efficiency of colonoscopy.

The mobile healthcare model established through the ColonClean app enables users to perceive the convenience and assistance of using their mobile devices combined with the mobile health app. The COVID-19 pandemic has altered individuals’ lifestyles, necessitating the maintenance of physical distancing from others to reduce virus transmission, once again highlighting the importance of mobile health apps. In the future, the diverse development trends of smart healthcare will aspire to use mobile health apps as unique tools for bowel preparation, aiming to achieve optimal bowel preparation quality and effectively reduce the incidence and mortality of colorectal cancer.

One approach to enhancing health service information in mobile healthcare models is to use mobile apps as a method of nursing guidance to assist patients. It is hoped that in the future, whether for outpatient cases, hospitalized patients, or those requiring bowel preparation, this approach can strengthen the effectiveness of bowel preparation education, enabling smooth completion of colonoscopies, avoiding interruptions, effectively reducing the workload on nursing staff, and decreasing medical costs.

## Figures and Tables

**Table 1:** Participants’ basic characteristics (*n* = 80).

Variable	All Samples (*n* = 80)	Group		*p ^a^*
		Experimental (*n* = 40)	Control (*n* = 40)	
Male, *n* (%)	41 (51.2)	17 (42.5)	24 (60)	0.12
Age (years), *n* (%)				0.58
20–30	2 (2.5)	2 (5.0)	0 (0.0)	
31–40	11 (13.8)	5 (12.5)	6 (15.0)	
41–50	17 (21.3)	6 (15.0)	11 (27.5)	
51–60	24 (30.0)	15 (37.5)	9 (22.5)	
61–70	10 (12.5)	7 (17.5)	3 (7.5)	
71–80	16 (20.0)	5 (12.5)	11 (27.5)	
BMI, mean ± SD	23.79 ± 3.97	22.58 ± 3.22	25.0 ± 4.72	0.02 *
Education level, *n* (%)				0.16
Elementary school	6 (7.5)	3 (7.5)	3 (7.5)	
Junior high school	8 (10.0)	2 (5.0)	6 (15.0)	
High school/vocational	17 (21.3)	6 (15.0)	11 (27.5)	
College/university	36 (45.0)	22 (55.0)	14 (35.0)	
Research institute or above	13 (16.3)	7 (17.5)	6 (15.0)	
Married, *n* (%)	61 (76.25)	31 (77.5)	30 (75.0)	0.23
Constipated, *n* (%)	17 (21.25)	10 (25)	7 (17.5)	0.27
Abdominal surgery, *n* (%)	28 (35.0)	13 (32.5)	15 (37.5)	0.64
Diabetes, *n* (%)	12 (15.0)	5 (12.5)	7 (17.5)	0.54
Family history of colorectal cancer, *n* (%)	14 (17.5)	7 (17.5)	7 (17.5)	1.00
Colonoscopy experience, *n* (%)	48 (60.0)	26 (65.0)	22 (55.0)	0.51

^a^ Comparison between the experimental and control groups. Note: *, *p* < 0.05.

**Table 2:** Frequency and percentage of endoscopists’ assessment of bowel preparation quality by group.

		Group				*p*
		Experimental		Control		
		*n*	%	*n*	%	
Endoscopists judgment of bowel preparation quality	Poor	0	0.0	7	17.5	0.016 *
	Fair	10	25.0	13	32.5	
	Good	26	65.0	19	47.5	
	Excellent	4	10.0	1	2.5	
Total		40	100.0	40	100.0	

Note: *, *p* < 0.05.

## Data Availability

The data presented in this study are available on request from the corresponding author. The data are not publicly available due to the ethics protocol of this study.
